# eHealth in the management of PTSD in children and adolescents: a scoping review

**DOI:** 10.3389/fpsyg.2026.1764838

**Published:** 2026-07-03

**Authors:** Liting Ju, Dan Zhang, Ruixin Guan, Yaokang Xue, Hairui Sun, Lili Hu, Baorong Zhang, Xiaoshuang Zhao

**Affiliations:** 1 Department of Pediatric Intensive Care Unit, The First Hospital of Jilin University, Changchun, Jilin, China; 2 Department of Nursing, The First Hospital of Jilin University, Changchun, Jilin, China

**Keywords:** child, adolescent, eHealth, PTSD, scoping review

## Abstract

**Background:**

Post-traumatic stress disorder (PTSD) in children and adolescents poses a significant public health challenge, particularly in settings with limited access to traditional mental health services. eHealth technologies are increasingly used to manage pediatric PTSD; however, evidence remains fragmented and unsynthesized.

**Aim:**

To comprehensively map the application of eHealth technologies in the management of PTSD among children and adolescents, with a focus on technology modalities, target populations, implementation settings, content elements, outcomes, feasibility, and acceptability. This review also aimed to identify current evidence gaps and highlight priorities for future research.

**Study design:**

Following the Arksey and O’Malley framework and PRISMA-ScR guidelines, seven databases were searched up to November 2025. Eligible studies involved eHealth tools targeting pediatric PTSD and included quantitative, qualitative, or mixed-methods designs. Study quality was appraised using the Mixed Methods Appraisal Tool (MMAT).

**Results:**

A total of 26 studies published between 2010 and 2025 were included. eHealth modalities encompassed websites, mobile applications, digital games, telemedicine, virtual reality, and wearable sensors, covering the full management continuum from training, assessment, prediction, prevention, intervention, to monitoring. Most studies focused on school-aged children and adolescents, with limited attention to preschoolers or vulnerable populations. Non-randomized studies generally reported short-term PTSD symptom improvements, whereas randomized controlled trials yielded inconsistent results, with limited long-term follow-up data. Overall, feasibility and acceptability were high; however, challenges included distractions in home environments, technical issues, privacy concerns, and variable adherence.

**Conclusion:**

eHealth demonstrates broad applicability and feasibility in pediatric PTSD management, yet evidence for clinical efficacy remains insufficient. The field is transitioning from “technology validation” toward “efficacy evaluation and system integration.” Future research should prioritize rigorous randomized controlled trials, extended follow-ups, culturally and developmentally appropriate designs, cost-effectiveness analyses, and deeper integration of eHealth into clinical care pathways. Policy support is essential to ensure sustainable implementation, especially in resource-limited settings.

## 1 Introduction

Exposure to traumatic events during childhood and adolescence represents a major public health concern. Estimates suggest that nearly 60% of children experience at least one traumatic event before reaching adolescence, placing them at heightened risk for a range of mental health problems (McLaughlin et al., 2013; Zhou et al., 2023). It has been reported that up to 30% of trauma-exposed children may develop PTSD symptoms, with approximately 10% receiving a formal diagnosis (Tamir et al., 2024). PTSD may manifest as nightmares, sleep disturbances, repetitive trauma-related thoughts or play, avoidance of trauma reminders, emotional distress, emotional numbing, and irritability (Long et al., 2022). A strong association has been identified between PTSD and long-term adverse outcomes, including depression, substance abuse, delinquency, and suicidal behavior, with enduring negative effects that may extend into adulthood (Zatzick et al., 2008; Ammerman et al., 2019). Therefore, early management of PTSD in children and adolescents is crucial.

However, the management of PTSD among children and adolescents faces multiple practical challenges. A critical barrier is the severe shortage and uneven geographic distribution of child mental health professionals, which substantially limits the timely delivery of assessment and intervention services (Anthes, 2016). This challenge is particularly pronounced in rural and resource-limited settings, where geographical distance, socioeconomic constraints, and underdeveloped mental health care systems further restrict access to psychological support (Graves et al., 2023). In addition, major public health emergencies or catastrophic events may disrupt the continuity of PTSD management. During the COVID-19 pandemic, for example, psychological needs related to PTSD among children and adolescents increased markedly, while face-to-face psychological interventions and follow-up services were significantly constrained (Yang et al., 2022). These challenges highlight the need to develop more flexible and scalable models for PTSD management in this population. eHealth technologies, such as telehealth-based service delivery, have been shown to effectively reduce barriers to accessing health care (Comer and Myers, 2016; Denecke et al., 2022).

eHealth has introduced novel opportunities for psychological management. It is defined as the use of information and communication technologies to support health care delivery (World Health Organization, 2025). Its technical features include: (i) enabling data storage, retrieval and transmission; (ii) supporting clinical decision-making; and (iii) facilitating remote care (Black et al., 2011). From a trauma-informed public health perspective, eHealth has been leveraged to implement essential interventions across the continuum of prevention, including primary, secondary, and tertiary levels, at both local and global scales (Magruder et al., 2016). Furthermore, such technologies are increasingly being used to assist individuals throughout the trauma timeline, including pre-trauma preparedness (e.g., through psychoeducation or resilience training) and post-trauma interventions (e.g., for assessment, early prevention, and evidence-based treatment) (Bakker et al., 2020).

The potential value of eHealth in the management of PTSD among children and adolescents can be interpreted through Engel’s biopsychosocial model (Engel, 1977). This model emphasizes the holistic nature of health and highlights the importance of integrating biological, psychological, and social factors in clinical practice to inform more comprehensive care strategies. At the biological level, wearable sensors and physiological monitoring technologies can facilitate the identification of objective stress-related responses. At the psychological level, eHealth interventions may improve PTSD-related outcomes through psychoeducation, emotion regulation training, and self-monitoring strategies. At the social level, eHealth technologies can enhance access to mental health services by leveraging the natural affinity of children and adolescents for digital technologies (McGar et al., 2019), thereby promoting family engagement and partially alleviating pressure on healthcare systems (Colasante et al., 2022).

Although previous studies have explored the application of eHealth in the management of PTSD among children and adolescents, several important limitations remain. Existing reviews have primarily examined specific technological modalities, trauma contexts, or adult populations, with relatively limited attention given to the broader application of eHealth across the continuum of pediatric PTSD management. For example, Schulte mainly focused on internet- and mobile-based psychological interventions for PTSD and did not systematically address other management components, such as assessment, prediction, prevention, and monitoring (Schulte et al., 2024). Similarly, McGar primarily examined eHealth interventions for psychological sequelae associated with physical illness and did not include PTSD related to non-medical traumatic exposures, such as violence or natural disasters, thereby overlooking a substantial proportion of trauma-exposed children and adolescents (McGar et al., 2019). In addition, previous reviews have predominantly emphasized quantitative evidence, whereas qualitative and mixed-methods studies have received comparatively less attention. However, the experiences and implementation feedback of children, adolescents, caregivers, and healthcare professionals are essential for optimizing the usability, acceptability, and contextual adaptability of eHealth tools. Neglecting such evidence may lead to a disconnect between technological development and real-world user needs. Collectively, these limitations hinder a comprehensive understanding of the technological landscape, application priorities, implementation characteristics, and evidence boundaries of eHealth in the management of PTSD among children and adolescents. They also constrain the evaluation of its clinical translation potential, implementation conditions, and future optimization strategies. Therefore, a scoping review is warranted to systematically map the existing evidence in this field. As a form of evidence synthesis, scoping reviews are particularly well suited to characterizing the breadth of evidence and identifying knowledge gaps within an emerging research area (Arksey and O’malley, 2005). Based on the limitations of previous reviews and the identified gaps in the existing evidence, this review addresses the following research questions: (i) What are the technology types and implementation settings of eHealth in the management of PTSD among children and adolescents? (ii) What are the characteristics of populations targeted by eHealth-based PTSD management? (iii) What management content elements and outcomes have been reported in existing eHealth applications? (iv) What evidence exists regarding the feasibility and acceptability of these approaches? and (v) What directions should guide future research and development in this field? It should also be noted that, although children and adolescents differ in developmental maturity, symptom presentation, and patterns of technology use, most existing studies have examined these populations together. Moreover, they share several characteristics relevant to this review, including reliance on caregiver support and the applicability of trauma-focused interventions. Including both populations therefore facilitates a more comprehensive understanding of how eHealth technologies may be adapted across pediatric developmental stages and helps identify current evidence coverage and application patterns at different stages of development.

## 2 Methods

This scoping review was conducted following the five-stage methodological framework proposed by Arksey and O’Malley (2005). The reporting adhered to the Preferred Reporting Items for Systematic Reviews and Meta-Analyses extension for Scoping Reviews (PRISMA-ScR) guidelines (Tricco et al., 2018). The review protocol was prospectively registered on the OSF platform.^1^

### 2.1 Search strategy

A preliminary search was carried out in PubMed and the Cochrane Library to identify existing reviews and avoid duplication. Comprehensive searches were subsequently performed in the following databases from their inception to November 2025: PubMed, Embase, Cochrane Library, Web of Science, CINAHL, PsycINFO, and Scopus. The search strategy incorporated both MeSH and relevant free-text terms, including “Child,” “PTSD,” and “eHealth.”(the full strategy is provided in Supplementary Table 1). An updated literature search was conducted in May 2026, and no additional eligible studies were identified. Grey literature was further retrieved through targeted searches in Google Scholar, review of reference lists from included studies and pertinent systematic reviews, as well as manual screening of potentially relevant records.

### 2.2 Study identification and inclusion/exclusion criteria

The eligibility criteria were developed according to the PCC framework. (1) Population (P): children and adolescents aged 0–18 years, their caregivers, or healthcare professionals. (2) Concept (C): eHealth technologies, classified according to the definition of eHealth described in the Introduction, including mobile applications, telehealth and videoconferencing, web-based platforms, virtual reality, digital games, and wearable technologies. Eligible studies were required to report PTSD-related clinical outcomes among children and adolescents based on the DSM-5 framework, or outcomes related to the acceptability, feasibility, or satisfaction associated with eHealth technologies. Quantitative, qualitative, and mixed-methods studies were included. (3) Context (C): healthcare or non-healthcare settings in which eHealth technologies were applied to the management of PTSD among children and adolescents. Studies published in languages other than English, studies without accessible full texts, reviews, conference abstracts, letters, study protocols, duplicate publications, and small-sample case reports (n ≤ 5) were excluded.

### 2.3 Study selection

All records were managed using EndNote version 21.0. Following the removal of duplicate entries, two reviewers (LJ and YX) independently conducted the initial screening of titles and abstracts based on the criteria outlined by Adams et al. (2022). The primary reviewer (LJ) examined all retrieved records, while a secondary reviewer (YX) independently evaluated a randomly selected subset comprising 10% of the total, demonstrating substantial inter-rater reliability (Cohen’s Kappa = 0.84; agreement rate = 96.47%). Full-text articles were subsequently reviewed in parallel by both reviewers, yielding an almost perfect level of agreement (Cohen’s Kappa = 0.98; agreement rate = 99.26%). Any discrepancies encountered during the screening process were resolved through discussion, with input from a senior researcher (XZ) serving as arbitrator when consensus could not be reached.

### 2.4 Data extraction

Data were independently extracted by two reviewers (LJ and YX) using standardized Excel forms. The extracted data included: (i) study characteristics (authors, publication year, country, study design, and theoretical framework); (ii) eHealth components (participant clinical profiles, technology types, operator training, functionality, and content elements); (iii) clinical outcomes, including PTSD assessment tools and key findings; (iv) feasibility and acceptability indicators; and (v) recommendations for future research. GraphPad Prism 9.5 was used to visualize the application phase distributions of different technological types.

### 2.5 Risk of Bias and quality assessment

To evaluate the methodological quality of the included studies, two reviewers (LJ and YX) independently applied the Mixed Methods Appraisal Tool (MMAT) (Hong et al., 2018). The tool comprises five quality criteria that assess the appropriateness of a study’s objectives, methodology, design, data collection, data analysis, presentation of findings, discussion, and conclusions. Each criterion was rated as either “Yes,” “No,” or “Unclear.” Any discrepancies in scoring were resolved through consensus discussions with adjudication by a third senior researcher (XZ) when necessary.

## 3 Results

### 3.1 Literature screening results

**Figure 1:**
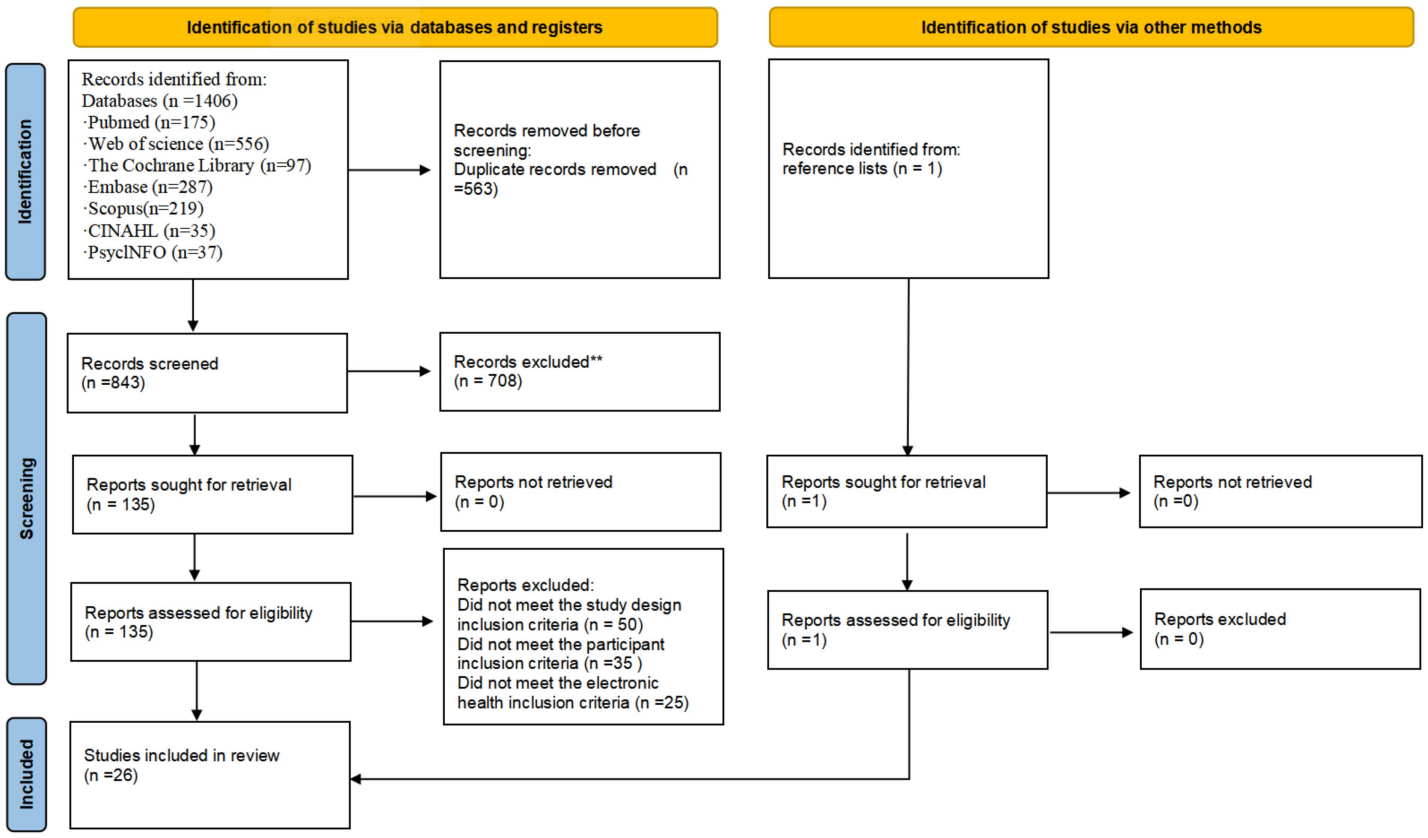
Flow chart.

The study selection process, depicted in the PRISMA flow diagram (Figure 1), began with the identification of 1,406 records retrieved from seven electronic databases. After the removal of 563 duplicates, 708 records were excluded based on title and abstract screening. Subsequently, 135 full-text articles were evaluated for eligibility. An additional study was identified through manual reference list screening. Ultimately, 26 studies met the inclusion criteria and were incorporated into the final synthesis.

### 3.2 Characteristics of included studies

All included studies were published between 2010 and 2025. Most studies originated from developed regions, including the United States (*n =* 18), followed by Iran (*n =* 2), Spain (*n =* 2), Australia (*n =* 1), the United Kingdom (*n =* 1), Norway (*n =* 1), and Palestine (*n =* 1). The majority of included studies employed non-randomized designs, including 10 quantitative non-randomized studies, 5 quantitative descriptive studies, and 4 mixed-methods studies, whereas only 7 studies were randomized controlled trials (RCTs). Only two studies explicitly reported the use of theoretical frameworks, namely the Behavioral Model of Health Services Use and the Cognitive Model of PTSD in conjunction with resiliency theory (Cox et al., 2010; Price et al., 2015). A comprehensive summary of study characteristics and key findings is presented in Table 1.

**Table 1:** Characteristics of the included studies.

Studies	Countries	Study design	Participants	Technological types	Function	Content elements	PTSD measurement	Results and findings
Price et al. (2015)	United States	RCT	2000 urban and rural adolescents (aged 12–17) affected by a tornado	Website	Intervention	Modular multimedia intervention incorporating trauma-focused psychoeducation, structured trauma exposure guidance, and evidence-based techniques to reduce avoidance behaviors and manage anxiety responses.	None	Overall intervention access rate was 35.8%, with module completion rates ranging from 52.8 to 85.6%.
Marsac et al. (2011)	United States	Quantitative non-randomized studies	50 parents of children (aged 6–17) who sustained injuries in the past 2 months	Website	Training/Prevention	Online psychoeducation featuring curated media on trauma responses, peer advice from other parents, and personalized care plans based on parent-rated symptom assessments.	None	Parents demonstrated consistently high knowledge of trauma responses (70% accuracy) before and after the intervention, and reported high satisfaction.
Piqueras et al. (2021)	Spain	Quantitative descriptive studies	1,499 children (aged 8–18) selected from eight primary and secondary schools	Website	Assessment	Online screening tool for assessing PTSD and other emotional disorders in children and adolescents.	CRIES	The tool demonstrates good reliability and validity, making it an effective instrument for mental health screening.
Jaycox et al. (2019)	United States	Quantitative non-randomized studies	51 middle and high school students from five schools	Website /Digital Game	Intervention	Evidence-based intervention integrating psychoeducation, emotion regulation, cognitive restructuring, and trauma narration, supported by interactive games and multimedia tools.	CPSS	The intervention was feasible and acceptable, with moderate satisfaction levels and notable PTSD reduction.
Omidvar Eshkalak et al. (2024)	Iran	Quantitative non-randomized studies	110 parents of children (aged 10–18) diagnosed with PTSD due to traumatic events	Website	Intervention	Four-week online stress management and therapy program for children and parents, featuring PTSD education, symptom recognition, and parental strategies for supporting recovery.	CRIES	Intervention group scored significantly lower than controls.
Kassam-Adams et al. (2019)	United States	Quantitative descriptive studies	167 children (aged 6–14) with illness or injury from two pediatric healthcare systems (urban and rural)	software system	Assessment	Avatar-based PTSD assessment conducted via mobile device.	CPSS-5	The tool was well accepted among school-aged children (9–14 years), with good preliminary reliability and validity.
Martin et al. (2023)	United States	Mixed- methods studies	46 foster children (aged 4–17) exposed to traumatic events with active trauma symptoms	Telemedicine	Intervention	TeleHealth-delivered TF-CBT	CATS	Remote therapy significantly reduced PTSD symptoms in children and adolescents, though adherence was suboptimal and completion barriers persisted.
Hashemi et al. (2017)	Palestine	Quantitative descriptive studies	986 children (aged 6–18) affected by war	software system	Assessment	Training social workers to use mobile applications (e.g., ODK) for mental health screening of war-affected children in conflict zones.	DSM-5 diagnostic criteria	Among war-affected children, high PTSD prevalence was observed; the app facilitated effective psychological screening in this population.
Stewart et al. (2020)	United States	Quantitative non-randomized studies	70 trauma-exposed youth (aged 7–18) and their caregivers	Videoconferencing/Digital game	Intervention	TeleHealth-delivered TF-CBT	UCLA-PTSD-RI	Post-treatment PTSD symptom reduction was clinically significant.
Heinz et al. (2022)	United States	Mixed- methods studies	7 children (aged 13–17) who lost homes or schools in the 2017 wildfires and reported ≥4 PTSD symptoms	App	Intervention/Monitoring	A 6-week, self-paced intervention with six modules combining evidence-based psychoeducation, audio guidance, and interactive components to support stress, emotion, and relationship management, featuring emotion-tracking visualizations, youth-specific content, and free connections to mental health care services.	CPSS-5	Due to the limited sample size, no significant effects were observed. However, based on engagement, satisfaction, and likelihood of recommending to friends, the tool appears to be feasible.
Wijesekera et al. (2023)	United States	RCT	17 pediatric heart transplant recipients (aged 8–18) and their parents	Videoconferencing	Prevention	Videoconferencing-based sessions including family-centered screening and feedback, psychoeducation, and training in family resilience skills.	CTSQ	No significant reduction in PTSS post-intervention, but acceptability and satisfaction were high.
Asnaani et al. (2021)	United States	Quantitative non-randomized studies	49 trauma-exposed children (aged 8–12)	Digital game	Assessment	Aquatic characters guide users to outline trauma types and screen for PTSD symptoms.	CPSS-5	The tool demonstrated strong reliability, validity, and acceptability for identifying children at risk of PTSD.
Hoover and Romero (2019)	United States	Quantitative descriptive studies	20 trauma-exposed children with autism spectrum disorder (aged 8–14)	App	Assessment	Touchscreen-based graphic interactive self-report.	UCLA-PTSD-RI	Among children with autism, the tool was feasible and acceptable, showing preliminary effectiveness in assessing trauma exposure and symptoms.
Villalobos et al. (2023)	United States	Mixed- methods studies	60 children with PTSD (aged 7–18) and their caregivers	videoconferencing	Intervention	Videoconferencing -delivered TF-CBT.	None	Adolescents and caregivers reported high levels of satisfaction and comfort.
Kassam-Adams et al. (2016)	United States	RCT	72 children (aged 8–12) hospitalized due to acute medical events	Digital game	Prevention	Story-driven interactive game guiding children through three levels to recognize emotions, adjust cognitions, and manage trauma cues via interactions with virtual characters representing diverse trauma experiences.	ASC-6; CPSS-5	The intervention was feasible, though effects were not statistically significant.
Stewart et al. (2017)	United States	Quantitative non-randomized studies	15 children (aged 7–16) with PTSD symptoms and their parents	Videoconferencing /Digital game	Intervention	Videoconferencing-delivered TF-CBT.	UCLA-PTSD-RI	PTSD symptoms in children and adolescents were significantly reduced, with a 100% adherence rate.
Goslin and Epstein (2024)	United States	Quantitative non-randomized studies	129 trauma-exposed children (aged 7–18) and their parents	Videoconferencing	Intervention/Training	A 5–8 session evidence-based brief intervention emphasizing trauma-informed care and family involvement to improve parent–child communication, shared trauma understanding, and collaborative coping, aiming to reduce child trauma symptoms and strengthen caregiver support.	CPSS-5	PTSS significantly decreased in both children and caregivers; caregiver satisfaction was high.
Marsac et al. (2013)	United States	RCT	100 parents of injured children (aged 6–17)	Website	Training/ prevention	Online psychoeducation featuring curated media on trauma responses, peer advice from other parents, and personalized care plans based on parent-rated symptom assessments.	PCL-C/PR; CPSS	The strategy was feasible but insufficient to improve child PTSD symptoms.
Hashemi et al. (2017)	Iran	Quantitative non-randomized studies	18 female youth soccer players (aged 12–13) who participated in an international tournament during the 2015 Nepal earthquake	Videoconferencing	Prevention	Remote online group and individual supportive psychotherapy.	CROPS; PROPS	Girls using this tool achieved stable psychological outcomes.
Shenk et al. (2022)	United States	RCT	33 children (aged 6–17) with PTSD symptoms following abuse or violence exposure	Sensor technology	Prediction	ECG-based RSA measurement for predicting pediatric PTSD.	UCLA-PTSD-RI; DSM-5 diagnostic criteria	Improved RSA regulation (i.e., lower RSA variability) was associated with reduced PTSD symptoms post-treatment.
Cox et al. (2010)	Australia	RCT	85 hospitalized children (aged 7–16) with accidental or unintentional injuries	Website	Prevention	Child-focused website offering self-help resources based on cognitive-behavioral and resilience-building strategies.	TSSCC-A	No significant intervention effects were observed; however, symptoms decreased in the intervention group and increased in the control group.
Fein et al. (2010)	United States	Quantitative descriptive studies	132 adolescents (aged 14–18) presenting to a tertiary pediatric emergency department for non-psychiatric complaints	software system	Assessment	After explanation by a nurse or technician, the child independently completes the screening using the system, which automatically generates reports for physician review. Positive cases are referred for social work or psychiatric assessment based on clinical judgment.	General psychiatric screening instruments	Following implementation of the screening tool, the proportion of adolescents identified with mental health issues increased significantly.
Smith et al. (2025)	United Kingdom	RCT	31 children (aged 12–17) who sought referral from health service centers and secondary schools in the same region, diagnosed with PTSD.	APP/ Website	Intervention	The program includes 11 core modules covering PTSD education, trauma narrative, trigger management, and coping skills, plus 11 optional modules addressing emotions like anger, shame, and guilt.	CPSS-5/CRIES/CAPS-CA-5	Therapist-supported online cognitive therapy for PTSD is acceptable to young people and shows potential for meaningful and sustained effects.
Prieto Larrocha et al. (2025)	Spain	Quantitative non-randomized studies	77 children and adolescents (aged 6–16) who experienced severe and chronic domestic violence were recruited.	VR	Intervention	Traditional TF-CBT was enhanced with the EMMA-Child VR system, incorporating psychoeducation, relaxation, emotion regulation, cognitive restructuring, VR-based trauma narrative, controlled exposure, and a self-concept reconstruction module.	CPSS	Significant improvement in child PTSD symptoms.
Birkeland et al. (2025)	Norway	Mixed- methods studies	59 adolescents (aged 13–18) with clinically significant trauma exposure and PTSS, along with their therapists.	APP	Intervention/ Monitoring	Psychoeducation on trauma, PTSD, and sleep; mental health self-monitoring illustrated with charts; a toolbox containing relaxation exercises, personalized strategies, a safe place, and positive cognitions; goal-setting features; and gamification elements.	CATS	This application may be useful for children receiving TF-CBT.
McDonnell et al. (2025)	United States	Quantitative non-randomized studies	17 autistic adolescents (aged 10–17) and their caregivers.	Telemedicine	Intervention	Videoconferencing-delivered TF-CBT.	ADIS-C; ADIS-P/CATS	Telehealth-delivered TF-CBT was feasible, effective, and acceptable for adolescents with autism.

ADIS-C, Anxiety Disorders Interview Schedule – Child Version; ADIS-P, Anxiety Disorders Interview Schedule – Parent Version; App, application; ASC-6, Acute Stress Checklist for Children–6-item Short Form; CATS, Child and Adolescent Trauma Screen; CAPS-CA-5, Clinician-Administered PTSD Scale for Children and Adolescents for DSM-5; CPSS, Child PTSD Symptom Scale; CPSS-5, Child PTSD Symptom Scale for Diagnostic and Statistical Manual of Mental Disorders, Fifth Edition; CRIES, Children’s Revised Impact of Event Scale; CROPS, Child Report of Posttraumatic Symptoms; CTSQ, Child Trauma Screening Questionnaire; DSM-5, Diagnostic and Statistical Manual of Mental Disorders, Fifth Edition; PCL-C/PR, PTSD Checklist for Children–Parent Report; PROPS, Parent Report of Posttraumatic Symptoms; PTSD, post-traumatic stress disorder; PTSS, Post-Traumatic Stress Symptoms; RCT, Randomized Controlled Trial; TF-CBT, Trauma-Focused Cognitive Behavioral Therapy; TSCC-A, Trauma Symptom Checklist for Children-A; UCLA-PTSD-RI, University of California at Los Angeles PTSD Reaction Index.

### 3.3 Quality assessment and risk of bias

Methodological quality assessment was conducted for all included studies. Overall, most studies demonstrated moderate to high methodological quality. Specifically, 2 studies achieved a quality score of 100%, 13 studies scored 80%, 8 studies scored 60%, 2 studies scored 40%, and 1 study scored 20%. Further details regarding the quality assessment are provided in the Supplementary Table 2.

### 3.4 Study populations

The included studies reported sample sizes ranging from 7 to 2,000 participants, with a cumulative total of 5,869 individuals. Among the 25 studies reporting age ranges, the widest range was from 4 to 17 years, while the narrowest was from 12 to 13 years. Most studies focused on school-aged children and adolescents, with only one study (Martin et al., 2023) addressing PTSD management in preschool-aged children (Table 2).

**Table 2:** Feasibility of eHealth.

Studies	Feasibility metrics	Measures	Feasibility outcomes	Reasons for dropout	Conclusion
Price et al. (2015)	Access rate; adherence rates	Module initiation; module completion (defined as reaching the final page)	35.8% accessed; 73.2% adherence	Busy schedule; privacy and safety concerns	Feasible among adolescents; strategies needed to enhance engagement.
Marsac et al. (2011)	Adherence rate	100% completion	96.20% adherence	Loss to follow-up	Feasible for parents.
Piqueras et al. (2021)	Not reported				
Jaycox et al. (2019)	Adherence rate; video viewing percentage; Number of chapters viewed	100% completion	Mean 78% adherence; students viewed 63–89% per chapter; mean chapters completed: 6.37 (SD = 1.3)	Dropout; disciplinary action	Good adherence and operational feasibility
Omidvar Eshkalak et al. (2024)	Adherence rate	100% completion	94.55% adherence	Voluntary withdrawal; non-cooperation	Feasible for parents.
Kassam-Adams et al. (2019)	Not reported				
Martin et al. (2023)	Adherence rate	100% completion	30.43% adherence	Placement/treatment changes; hybrid delivery	Feasible; barriers to adherence remain.
Hashemi et al. (2017)	Not reported				
Stewart et al. (2020)	Adherence rate; safety; technical performance	100% completion; Safety incidents; technical issues	88.6% adherence; no safety issues; initial login problems, occasional lag	Not reported	Highly feasible; stable internet needed.
Heinz et al. (2022)	Adherence rate	100% completion	85.71%	Download difficulties	Feasibility demonstrated by user engagement.
Wijesekera et al. (2023)	Adherence rate	100% completion	66.70%	Medical/scheduling issues	Preliminary feasibility for transplant families.
Asnaani et al. (2021)	Not reported				
Hoover and Romero (2019)	Not reported				
Villalobos et al. (2023)	Not reported				
Kassam-Adams et al. (2016)	Intervention usage; adherence rate; number of uses Total usage time	Platform login; completed all levels; number of logins total usage time	Immediate Intervention Group: 97% completed initial login; 53% completed the entire intervention; mean number of logins: 2.6; mean total usage time: 52.2 min. Waitlist Control Group: 68% logged into the intervention after 12 weeks; 54% completed the entire intervention; mean number of logins: 2.7; mean total usage time: 51.5 min.	One child unwell; others unreported	Feasible for post-medical trauma.
Stewart et al. (2017)	Technical performance; safety; treatment completion frequency; adherence rate.	Technical issues; safety incidents; 100% completion	Minimal technical issues (initial login problems, brief pixelation); no safety concerns; average of 14.13 sessions; 100% adherence	Not reported	High completion rates, minimal technical and safety issues; strong overall feasibility.
Goslin and Epstein (2024)	Not reported				
Marsac et al. (2013)	Adherence rate	100% completion	100% inpatient adherence; 56% parents continued post-discharge	Not reported	Feasible during hospitalization; moderate long-term adherence requires support.
Hashemi et al. (2017)	Not reported				
Shenk et al. (2022)	Not reported				
Cox et al. (2010)	Adherence rate	Website access	56.30% adherence	Unable to contact, too busy; Perceived lack of need for intervention	Moderately feasible for pediatric injury cases.
Fein et al. (2010)	Adherence rate	screening completion	64.60% adherence	Declined participation	Feasible within pediatric ED workflow.
Smith et al. (2025)	1. Number of referrals and their sources 2. Number of assessment appointments offered, number and proportion of completed assessments 3. Proportion of adolescents meeting eligibility criteria after assessment 4. Number and proportion retained at 16-week treatment and 38-week follow-up 5. Number of system logins (weekly and total) 6. Duration of system use (weekly and total) 7. Number of modules completed (total and by device type) 8. Number of calls with therapist (weekly/total) and missed calls 9. Duration of phone calls (weekly/total) 10. Adverse events		1. 78% of referrals and 87% of participants from CAMHS 2. 73 assessment appointments offered; 62 (85%) attended 3. 58% (36/62) eligible; 89% (32/36) consented; 97% (31/32) randomized 4. ICT-PTSD-YP: 100% (16/16) completed 16-week data, 94% (15/16) at 38 weeks; WL: 87% (13/15) completed 16-week data 5. Participants logged in about once a week on average. They logged in more often (about twice a week) during the first 3 weeks, then rarely logged in (median zero times) in the last 3 weeks. 6. Weekly login duration: 4 h (weeks 1–2), 1 h (weeks 3–10), median 0 (weeks 11–16) 7. Median 9 (IQR 2.8) modules completed; more early (weeks 0–3), fewer later (weeks 10–16) 8. Median 21 (IQR 2.2) therapist calls over 16 weeks; median 2 calls weeks 1–4 and week 16 9. Weekly call duration median 33 min (IQR 4.4) 10. 2 serious adverse events unrelated to study; 7 adverse reactions, mostly temporary PTSD symptom increases after memory modules	Participation in non-experimental psychotherapy; declined or incomplete online questionnaires	Met all predetermined feasibility criteria, including successful recruitment, participant retention, and data collection.
Prieto Larrocha et al. (2025)	Adherence rate	100% Completion of intervention and post-intervention assessment	25.99% adherence	Return to biological family, adoption, foster care, or change of region/country	Experienced a relatively high sample attrition rate.
Birkeland et al. (2025)	Adherence rate; safety	100% Completion; Adverse events	56.67% adherence; None	No response; child did not consent to continue participation	Feasible for adolescents receiving TF-CBT.
McDonnell et al. (2025)	Adherence rate	100% Completion	100% adherence	Not reported	Feasible for adolescents with autism.

ED, Emergency Department.

The included studies targeted diverse populations and trauma contexts related to pediatric PTSD management. Overall, most studies focused on children and adolescents at elevated risk for PTSD or those with clinically significant trauma-related symptoms. 14 studies focused on children at high risk for PTSD, 10 investigated children who had a formal PTSD diagnosis, met symptom criteria, or presented with other psychological problems, and 2 examined school-based populations. The children involved had diverse trauma exposures, including natural disasters (*n =* 5), war (*n =* 2), witnessed or experienced abuse or violence (*n =* 9), illness or injury (*n =* 12), bereavement following the loss of close family or friends (*n =* 6), robbery (*n =* 3), sexual assault (*n =* 2) and vulnerable populations such as children with autism or those in foster care. 22 studies involved child participants, while 8 included parental components. Healthcare provider training was implemented in 11 studies, 2 of which employed videoconferencing for virtual training (Goslin and Epstein, 2024; McDonnell et al., 2025).

### 3.5 Modalities and application stages

**Figure 2:**
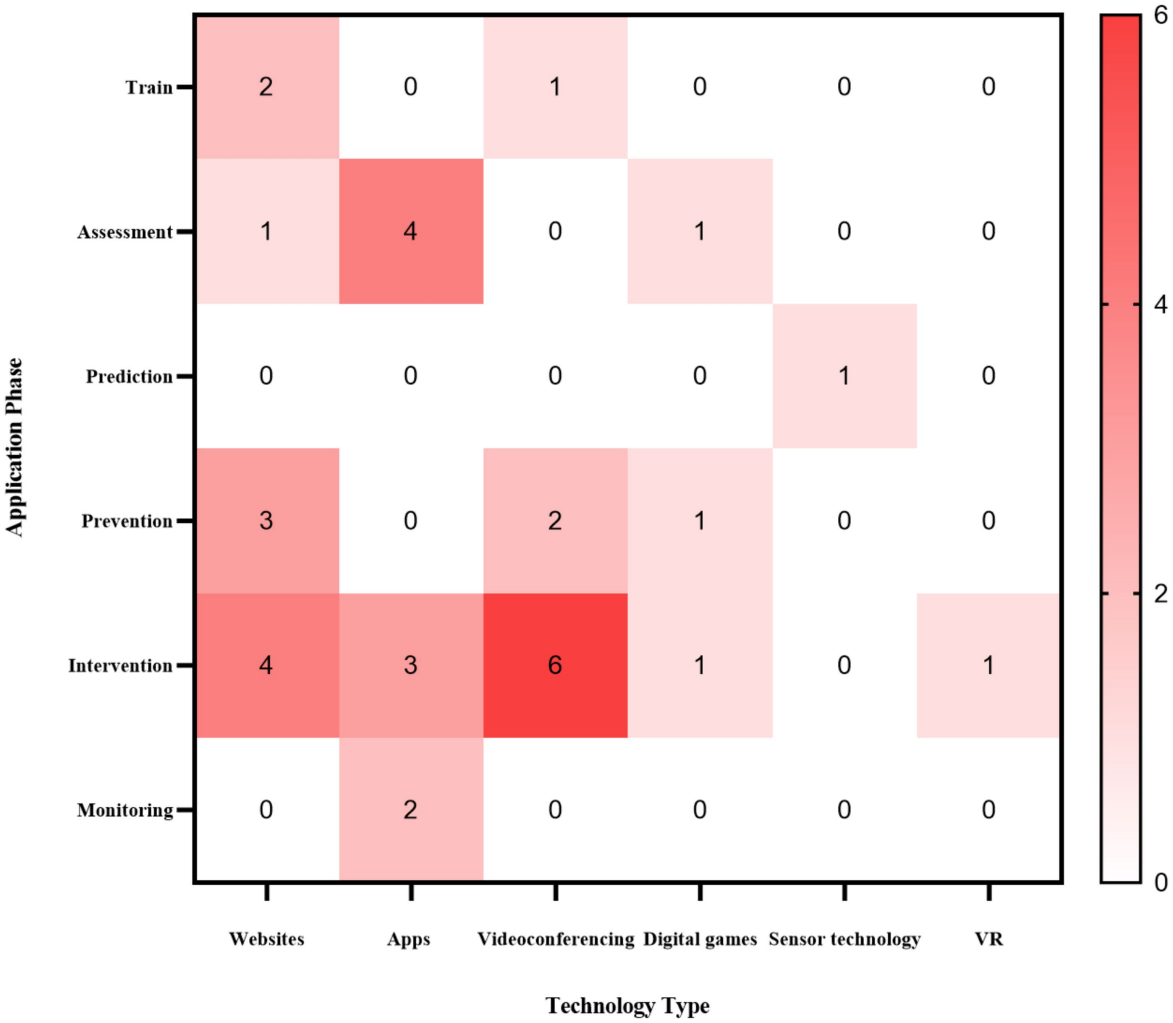
Heatmap of application stages across technology types.

The eHealth modalities identified in the included studies primarily comprised web-based platforms, digital games, mobile applications and software, videoconferencing systems, virtual reality, and wearable or sensor-based technologies. Current applications of eHealth in the management of PTSD among children and adolescents span multiple stages of care; however, existing research has predominantly focused on the intervention stage. Specifically, eHealth technologies were applied in training (*n =* 3), assessment (*n =* 6), monitoring (*n =* 2), prediction (*n =* 1), prevention (*n =* 5), and intervention (*n =* 14). A heatmap illustrating the distribution of different eHealth modalities across application stages is presented in Figure 2.

### 3.6 Implementation settings and delivery approaches

The implementation settings for eHealth in PTSD management encompassed both healthcare and non-healthcare environments. 8 studies were conducted in healthcare settings, including emergency departments (Fein et al., 2010; Kassam-Adams et al., 2019), pediatric clinics (Hoover and Romero, 2019; Asnaani et al., 2021), inpatient units (Kassam-Adams et al., 2016; Kassam-Adams et al., 2019), mental health departments (Asnaani et al., 2021; Shenk et al., 2022), and trauma centers (Marsac et al., 2013). One intervention adopted a “hospital-initiated, home-continued” model (Kassam-Adams et al., 2016). Several other studies implemented child PTSD management strategies in non-healthcare settings, such as homes, schools, and communities. These studies emphasized self-directed participation by children and their families (Cox et al., 2010; Marsac et al., 2011; Price et al., 2015; Jaycox et al., 2019; Piqueras et al., 2021; Heinz et al., 2022; Goslin and Epstein, 2024; Omidvar Eshkalak et al., 2024; Birkeland et al., 2025), as well as therapist- or physician-led telemedicine interventions (Hassanmirzaei et al., 2017; Stewart et al., 2017; Kassam-Adams et al., 2019; Stewart et al., 2020; Villalobos et al., 2023; Wijesekera et al., 2023; McDonnell et al., 2025; Smith et al., 2025). One study deployed eHealth screening in a post-conflict region, where trained personnel conducted on-site mental health assessments of children (Hashemi et al., 2017).

### 3.7 Assessment tools for pediatric PTSD

Various standardized instruments were used to assess PTSD-related symptoms among children and adolescents across the included studies. These included the Children’s Revised Impact of Event Scale (CRIES); the Child PTSD Symptom Scale (CPSS); the Child PTSD Symptom Scale for Diagnostic and Statistical Manual of Mental Disorders, Fifth Edition (CPSS-5; DSM-5); the Child and Adolescent Trauma Screen (CATS); and the University of California at Los Angeles PTSD Reaction Index (UCLA-PTSD-RI). Additional tools comprised the Child Trauma Screening Questionnaire (CTSQ); the PTSD Checklist for Children–Parent Report (PCL-C/PR); the Child Report of Posttraumatic Symptoms (CROPS) and the Parent Report of Posttraumatic Symptoms (PROPS); and the Trauma Symptom Checklist for Children-A (TSCC-A). The Acute Stress Checklist for Children–6-item Short Form (ASC-6), Clinician-Administered PTSD Scale for Children and Adolescents for DSM-5 (CAPS-CA-5); Anxiety Disorders Interview Schedule – Child/Parent Version (ADIS-C/P); general psychiatric screening instruments; and DSM-5 diagnostic criteria were also utilized in specific studies.

### 3.8 Content elements

#### 3.8.1 Web-based platforms

A total of eight studies utilized web-based platforms as the primary delivery modality, highlighting their broad accessibility and capacity for self-directed management. These platforms were mainly applied to PTSD symptom screening, psychoeducation, family support, intervention guidance, personalized care, and social problem-solving support. For example, Piqueras et al. (2021) developed the Detecta Web–Distress Scale, an internet-based self-assessment tool designed for early PTSD screening among school populations. Omidvar Eshkalak et al. (2024) and Smith et al. (2025) used website-based modules to deliver self-guided training sessions. Price et al. (2015), Jaycox et al. (2019), and Smith et al. (2025) provided trauma-related knowledge, emotion-regulation skills, and recovery strategies to children through multimodal website materials incorporating text, graphics, animations, and videos.

#### 3.8.2 Telemedicine or videoconferencing

A total of eight studies were implemented through telemedicine or videoconferencing to provide evidence-based PTSD interventions. Most studies were grounded in trauma-focused cognitive behavioral therapy (TF-CBT), supporting the delivery of standardized psychological interventions to children and their caregivers in non-clinical settings. Hashemi et al. (2017) also addressed social-domain interventions, including the restoration of social functioning and the enhancement of interpersonal support among affected children. Interventions comprised a fixed format of 12–17 sessions across studies (Stewart et al., 2017; Stewart et al., 2020; Martin et al., 2023; Villalobos et al., 2023; McDonnell et al., 2025). Goslin and Epstein (2024) implemented brief evidence-based trauma interventions for children and families, typically comprising 5–8 sessions. Hashemi et al. (2017) implemented a multi-phase schedule, providing intensive support through daily contacts during the initial phase, followed by weekly or monthly maintenance sessions for ongoing follow-up (Hassanmirzaei et al., 2017).

#### 3.8.3 Digital games

Five studies employed digital games to support the management of PTSD in children. Asnaani et al. (2021) gamified trauma screening by embedding standardized assessment tools within an aquatic-themed character environment. Other interventions were developed based on TF-CBT frameworks (Jaycox et al., 2019; Stewart et al., 2020) or incorporated adventure-game mechanics (Kassam-Adams et al., 2016; Jaycox et al., 2019). Stewart et al. (2020) developed the game using commonly available software tools such as Microsoft PowerPoint, Word, Excel, and Adobe Acrobat, ensuring strong platform compatibility and flexible deployment (Stewart et al., 2017; Stewart et al., 2020). Kassam-Adams et al. (2016) utilized an interactive narrative game in which children assisted virtual characters experiencing various traumas, enabling them to learn and practice emotion recognition, cognitive restructuring, and adaptive coping skills.

#### 3.8.4 Apps or systems

Seven studies applied mobile applications or systems to the assessment, monitoring, and intervention of PTSD. In hospital-based settings, three studies primarily focused on virtual character technologies (Kassam-Adams et al., 2019) and graphical interactive mechanisms (Hoover and Romero, 2019; Smith et al., 2025) to enhance children’s engagement and autonomy. Fein et al. (2010) further developed a digital assessment workflow guided by healthcare professionals, in which children independently completed assessments and reports were automatically generated by the system, thereby enabling standardized and efficient screening in emergency department settings. In out-of-hospital and resource-limited settings, Hashemi et al. (2017) trained social workers to use a mobile application to conduct rapid psychological screening and remotely upload data for trauma-affected children in war-affected regions. In addition, Heinz et al. (2022) and Birkeland et al. (2025) employed mobile applications for daily or weekly self-reported monitoring of PTSD symptoms among children. Furthermore, Heinz et al. (2022), Birkeland et al. (2025), and Smith et al. (2025) incorporated psychoeducation-based self-guided intervention modules to provide symptom management and psychological support for children. Notably, the study by Heinz et al. (2022) also included components addressing children’s descriptions and interpretations of others’ behaviors at the social level.

#### 3.8.5 VR

Prieto Larrocha et al. (2025) implemented an interactive virtual environment that enabled patients to regulate emotions through selectable symbolic images or music, reconstruct trauma narratives within a virtual “Book of Life,” and modify traumatic memories using a meaning-conversion tool.

#### 3.8.6 Sensor technology

Shenk et al. (2022) employed dynamic three-lead electrocardiography (ECG) monitoring to assess respiratory sinus arrhythmia (RSA) following trauma-focused cognitive behavioral therapy (TF-CBT). From a physiological perspective, their findings indicated that RSA regulation may serve as a predictor of PTSD symptom improvement.

### 3.9 Outcomes of eHealth interventions

The outcomes assessed across the 26 included studies covered three main domains: caregivers’ knowledge of PTSD, improvement in PTSD symptoms among children and adolescents, and PTSD symptoms in caregivers.

#### 3.9.1 Knowledge level

Two quantitative non-randomized studies evaluated the impact of eHealth interventions on caregivers’ PTSD-related knowledge. One study found that parents demonstrated a consistently high level of knowledge regarding psychological harm reactions (70% accuracy), with no intervention-related improvement (Marsac et al., 2011). The other reported immediate post-intervention gains that were not maintained at the 6-week follow-up (Marsac et al., 2013).

#### 3.9.2 Child PTSD symptoms

A total of 16 studies evaluated the effects of eHealth interventions on children’s PTSD symptoms. Among the five RCTs, one study reported that the eHealth intervention reduced the probability of meeting PTSD diagnostic criteria by 80% at 16 weeks compared with the waitlist group, with significant symptom reductions sustained to 38 weeks (Smith et al., 2025). The remaining four RCTs found no significant effects across follow-up periods ranging from 4 weeks to 6 months (Cox et al., 2010; Marsac et al., 2013; Kassam-Adams et al., 2016; Wijesekera et al., 2023). A total of 11 studies used non-randomized designs. 7 of these reported significant symptom reductions immediately after the intervention (Stewart et al., 2017; Jaycox et al., 2019; Stewart et al., 2020; Martin et al., 2023; Goslin and Epstein, 2024; Birkeland et al., 2025; Prieto Larrocha et al., 2025), and two additional studies observed significant improvements at 1-month and 6-week follow-up, respectively (Omidvar Eshkalak et al., 2024; McDonnell et al., 2025). These effects were primarily short-term. One study found no significant short-term impact (Heinz et al., 2022). Another reported decreases in both child- and parent-reported PTSD scores and noted that online supportive psychotherapy was positively associated with symptom reduction; although symptoms resolved completely among high-risk children, no statistical significance testing was conducted (Hassanmirzaei et al., 2017).

Therapist-guided telehealth interventions, such as TF-CBT, generally reported stable and significant reductions in PTSD symptoms. In contrast, findings for fully self-guided digital interventions, including web-based programs and self-help applications, were inconsistent. Although a small number of randomized controlled trials reported significant benefits, most studies failed to demonstrate superiority over control conditions on core PTSD outcomes.

#### 3.9.3 Caregiver PTSD symptoms

Five studies assessed caregivers’ PTSD symptoms. Across 3 RCTs, no significant intervention effects on parental PTSD symptoms were detected at follow-up periods ranging from 6 weeks to 6 months (Cox et al., 2010; Marsac et al., 2013; Wijesekera et al., 2023). In contrast, two non-randomized studies reported significant short-term improvements, with effects observed immediately post-intervention and up to 1 month (Goslin and Epstein, 2024; McDonnell et al., 2025).

### 3.10 Feasibility and acceptability of eHealth interventions

#### 3.10.1 Feasibility

A total of 17 studies assessed the feasibility of eHealth. Although assessment methods varied, all studies used adherence rates as a primary feasibility indicator. Nine studies reported high adherence (>75%), including 2 with full adherence (100%). Six studies showed moderate adherence (50–75%), while two studies reported low adherence (25.99 and 30.43%). Additional indicators included access rates (Price et al., 2015; Kassam-Adams et al., 2016), video completion percentages (Jaycox et al., 2019), the number of video chapters viewed (Jaycox et al., 2019; Smith et al., 2025), usage frequency and duration (Kassam-Adams et al., 2016; Smith et al., 2025), safety (Stewart et al., 2017; Stewart et al., 2020; Birkeland et al., 2025; Smith et al., 2025), and technical performance (Stewart et al., 2017; Stewart et al., 2020).

#### 3.10.2 Acceptability

A total of 15 studies evaluated the acceptability of eHealth, primarily through satisfaction questionnaires and qualitative feedback on user perceptions. Eight studies demonstrated participant satisfaction scores consistently ranging within the moderate-to-high range. Participants commonly cited ease of use (Price et al., 2015; Stewart et al., 2017; Kassam-Adams et al., 2019; Heinz et al., 2022; Birkeland et al., 2025), willingness to recommend to others (Price et al., 2015; Stewart et al., 2017; Kassam-Adams et al., 2019; Heinz et al., 2022; Goslin and Epstein, 2024), intention to continue using it (Marsac et al., 2011), perceived helpfulness (Cox et al., 2010; Hoover and Romero, 2019; Kassam-Adams et al., 2019; Heinz et al., 2022; Goslin and Epstein, 2024; Birkeland et al., 2025), comprehensibility (Hoover and Romero, 2019; Asnaani et al., 2021; Birkeland et al., 2025), a strong therapeutic alliance (Stewart et al., 2017), a positive user experience (Hoover and Romero, 2019), minimal privacy concerns (Villalobos et al., 2023), and scheduling flexibility and reduced travel burden (Villalobos et al., 2023; Wijesekera et al., 2023; Birkeland et al., 2025). Reported barriers included difficulties maintaining children’s attention at home; lack of privacy; concerns about the authenticity and completeness of non–face-to-face assessments; disruptions when caregivers had to reschedule sessions in public settings; therapy interruptions due to relocations; psychological distress triggered by confronting adverse self-reported results; technical issues such as login failures and internet instability; and burden associated with the large number of questionnaires, some of which were perceived as overly predictable (Stewart et al., 2017; Stewart et al., 2020; Martin et al., 2023; Wijesekera et al., 2023; Birkeland et al., 2025). The results for feasibility are presented in Table 2, while those for acceptability are provided in Table 3.

**Table 3:** Acceptability of eHealth.

Studies	Acceptability measures	Key findings	Facilitators	Barriers
Price et al. (2015)	4-month follow-up interviews	76.43% found the website easy to use; 73.93% would recommend it	Ease of use	Not reported
Marsac et al. (2011)	Parent Satisfaction Questionnaire	High parental satisfaction; reuse higher in web group (76%) vs. video (44%)	Not reported	Not reported
Piqueras et al. (2021)	Not reported			
Jaycox et al. (2019)	Satisfaction rating (scale: 0–3)	Moderate satisfaction(M = 1.85); variation by school grade level	Not reported	Not reported
Omidvar Eshkalak et al. (2024)	Not reported	Free access may have enhanced acceptance	Free access	Not reported
Kassam-Adams et al. (2019)	TAM	Children found it helpful and easy to use; lower usability in ages 6–8	Ease of use	Younger children usability issues
Martin et al. (2023)	Focus groups	Acceptability limited by systemic, technical, and family-related barriers	Flexible scheduling	Inattention, unstable internet, lack of privacy at home, Assessments relied on caregiver self-report, requiring 8 rescheduling when caregivers were in public settings. Frequent treatment disruptions also occurred due to placement instability in the foster care system.
Hashemi et al. (2017)	Not reported			
Stewart et al. (2020)	Not reported	Not reported	Not reported	Login issues, internet instability
Heinz et al. (2022)	Satisfaction rating (0 = very dissatisfied to 10 = very satisfied)	High satisfaction (M = 8.50); found helpful, functional, and easy to use; would recommend it	Ease of use, helpful content	Not reported
Wijesekera et al. (2023)	FOCUS-PedsHT acceptability and satisfaction scales	Adolescents and parents gave positive feedback; four teens reported difficulty focusing at home	Flexible scheduling	Difficulty concentrating at home
Asnaani et al. (2021)	Qualitative feedback	Participants rated the intervention highly for feasibility, clarity, and enjoyment	Easy to understand; enjoyable design	Not reported
Hoover and Romero (2019)	User satisfaction questionnaire	Highly positive ITS ratings across all domains; 74% found it comfortable and easy to understand, 76% said it helped with emotion recognition, 88% had positive overall experience	Easy to understand	Not reported
Villalobos et al. (2023)	TSQ	High satisfaction from children and caregivers; 100% felt respected by therapists; >95% preferred telehealth over in-person; no privacy concerns reported	Flexible scheduling at home/school; Eliminated commuting burden;privacy protection	Not reported
Kassam-Adams et al. (2016)	Not reported			
Stewart et al. (2017)	TSQ and semi-structured interviews	100% of caregivers reported satisfaction; providers also highly satisfied; 86% found the platform easy to use; all reported strong therapeutic alliance and would recommend it Both caregivers and children reported feeling comfortable communicating via telehealth	Good therapeutic relationship; Ease of use; convenience; comfort	Login difficulties, unstable internet
Goslin and Epstein (2024)	Caregiver satisfaction questionnaire	98% found it helpful; 96% felt better able to support child; 94.4% would recommend it	Not reported	Not reported
Marsac et al. (2013)	Not reported			
Hashemi et al. (2017)	Not reported			
Shenk et al. (2022)	Not reported			
Cox et al. (2010)	Subjective intervention evaluation questionnaire	63% of children and 56% of parents found it helpful; fewer rated it effective; “helpful” and “effective” ratings were significantly correlated	Not reported	Not reported
Fein et al. (2010)	Not reported			
Prieto Larrocha et al. (2025)	Not reported			
Birkeland et al. (2025)	Not reported			
McDonnell et al. (2025)	Qualitative interviews	Participants perceived several modules as useful, easy to understand, relevant, supportive of self-understanding, easy to follow, convenient, and motivating for children.	Useful, easy to understand, relevant, supportive of self-understanding, easy to follow, convenient,	Daily questionnaires contained too many items and were perceived as boring; questions were predictable; unfavorable self-assessment results induced guilt; privacy concerns were noted.
Prieto Larrocha et al. (2025)	Treatment Satisfaction and Feedback Measures; TSQ	Adolescents reported high acceptability, with all scores >3.5/5, indicating positive perceptions of enjoyment, comprehension, helpfulness, recommendation likelihood, agreement with content, and engagement. And caregivers rated all items ≥4.5. Adolescents found the relaxation, emotion, and safety modules easiest to understand and most helpful, with the interoception module rated lowest (3.20). Caregivers rated psychoeducation, parenting, and safety modules highest in comprehension and identified psychoeducation, emotion, and joint modules as most helpful; only comprehension of the joint module was below 4.0 (3.88). Both groups reported high satisfaction with telehealth delivery.	Useful, easy to understand	Not reported

TAM, Technology Acceptance Measure; TSQ, Telehealth Satisfaction Questionnaire.

### 3.11 Recommendations for future research

Among the included studies, 25 studies explicitly proposed future directions or recommendations for the development of eHealth targeting pediatric PTSD. Four studies focused on improving and optimizing the technological platform, including adding interpretive summaries for post-screening clinical results, incorporating gamified design elements, and enhancing system adaptability (Fein et al., 2010; Kassam-Adams et al., 2016; Asnaani et al., 2021; Birkeland et al., 2025). Five studies emphasized tailored interventions for specific target populations, including adapting content and delivery formats for younger children or those at higher risk (Cox et al., 2010; Kassam-Adams et al., 2016; Martin et al., 2023; Wijesekera et al., 2023; McDonnell et al., 2025). A total of 21 studies emphasized the need to optimize intervention timing, frequency, and study design, including the use of stepped-care models, the incorporation of appropriate control groups, the enlargement of sample sizes, the conduct of high-quality randomized controlled trials, the extension of follow-up durations, and the further validation of assessment tools. In addition, six studies addressed policy-level considerations, including streamlined approval processes by post-disaster regulatory authorities, universal broadband access, integration of telehealth costs into insurance policies, and the long-term sustainability of public programs (Fein et al., 2010; Price et al., 2015; Kassam-Adams et al., 2019; Stewart et al., 2020; Heinz et al., 2022; Goslin and Epstein, 2024). One study recommended expanding eHealth-related skills training for healthcare professionals (McDonnell et al., 2025).

## 4 Discussion

This study represents the first scoping review to map the application of eHealth technologies in the management of PTSD among children and adolescents. Existing evidence suggests that early studies (2010–2015) primarily focused on relatively simple web-based platforms and mobile applications, whereas from 2016 onward, eHealth technologies progressively evolved toward more interactive and diversified technological approaches. Since 2020, the COVID-19 pandemic has further accelerated the adoption and sustained use of telehealth-based interventions (Conradi et al., 2022). Overall, eHealth technologies have been applied across the full continuum of PTSD management, including training, assessment, prediction, prevention, intervention, and monitoring, and have been implemented in a wide range of settings, from emergency departments and outpatient clinics to homes, communities, schools, and post-conflict regions. However, the evidence base remains unevenly distributed. Most studies have focused on intervention-related applications, while evidence regarding prediction, long-term monitoring, and training remains relatively limited. In addition, the majority of studies have concentrated on school-aged children and adolescents, with insufficient evidence for preschool children and other vulnerable subgroups. Most non-randomized studies reported short-term improvements in PTSD symptoms among children and adolescents, whereas findings from RCTs were inconsistent. Moreover, the methodological quality of the included studies varied, with a considerable proportion being exploratory or feasibility-oriented, often characterized by small sample sizes and limited long-term follow-up. Therefore, current evidence on the real-world clinical effectiveness of eHealth interventions for pediatric PTSD should be interpreted with caution. Although feasibility and acceptability are well supported, implementation continues to face challenges related to attention demands, technical stability, and privacy protection.

### 4.1 Analysis based on the biopsychosocial framework

To interpret the above findings, this review synthesizes the existing evidence through the biopsychosocial framework.

#### 4.1.1 Biological level: potential and challenges of objective monitoring

At the biological level, wearable sensors have advanced PTSD monitoring and prediction by shifting the paradigm from reliance on children’s subjective recall toward the integration of objective physiological indicators. Their core value lies in capturing latent autonomic nervous system following trauma exposure, thereby addressing limitations of traditional self-report measures, which may be influenced by developmental stage, shame-related response bias, or memory distortion (Acheson et al., 2014). However, this field remains in an early stage of development and faces two major challenges. First, in non-clinical settings, sensor data quality is highly susceptible to motion artifacts, device displacement, and poor adherence, which may compromise data reliability in real-world contexts. Second, commonly used physiological indicators such as heart rate exhibit limited specificity for PTSD and may be difficult to distinguish from normal physical activity, generalized anxiety, or somatic discomfort. Therefore, the validity and stability of this technology in complex real-world environments require further systematic evaluation.

#### 4.1.2 Psychological level: discrepancy between evidence and expectation

From a psychological perspective, eHealth interventions may improve PTSD-related outcomes through mechanisms such as psychoeducation, emotion regulation training, self-monitoring, and enhanced treatment engagement. However, current evidence regarding effectiveness remains limited. Consistent with the meta-analysis by Schulte et al. (2024), this review similarly found that existing randomized controlled trials have not demonstrated stable or consistent positive effects: among the five RCTs included, only one reported sustained symptom improvement up to 38 weeks. In contrast, non-randomized studies more frequently reported short-term reductions in PTSD symptoms. Nevertheless, interpretation of these differences should be approached with caution. The positive findings from non-randomized studies may be influenced by selection bias, absence of active controls, natural recovery trajectories, and short follow-up periods, whereas inconsistent RCT findings may be attributable to limited sample sizes, variability in intervention adherence, and challenges associated with home-based implementation. Notably, therapist-guided remote trauma-focused interventions (e.g., telehealth-delivered TF-CBT) still demonstrate preliminary promise, potentially due to therapists’ ability to ensure treatment fidelity, adapt intervention strategies in real time, and establish therapeutic alliance in remote settings, functions that are difficult to replicate in fully automated interventions. Although meta-analyses in adult PTSD populations have shown that telehealth interventions may be comparable to face-to-face therapy (Scott et al., 2022), children and adolescents differ in developmental stage, caregiver dependence, and emotional expression patterns. Therefore, evidence derived from adult populations cannot be directly generalized to pediatric settings. At present, eHealth interventions should be considered as complementary approaches that enhance service accessibility, extend continuity of care, and support ongoing psychological management, rather than as standalone replacements for face-to-face treatment (Sethi, 2013; Vigerland et al., 2016). Furthermore, in the management of PTSD among children and adolescents, caregivers are not merely accompanying participants, but play a critical role in treatment engagement, home-based implementation, and maintenance of intervention effects. This role may be particularly important in eHealth interventions, where many services are delivered outside traditional clinical settings and caregivers often influence whether children can successfully access, engage in, and complete interventions. In addition, caregivers themselves may experience trauma-related psychological distress following a child’s traumatic exposure, and their psychological status may subsequently affect the child’s recovery process and intervention outcomes. However, caregiver-related factors remain insufficiently incorporated into current effectiveness evaluations.

Different eHealth modalities demonstrate distinct functional profiles. Web-based platforms, characterized by low cost and broad accessibility, play an important role in large-scale psychoeducation and information dissemination (Chiauzzi et al., 2008; Andersson et al., 2011), although their predominantly one-way delivery format may limit the depth of intervention engagement. Videoconferencing and telehealth modalities are more suitable for therapist-guided structured interventions, such as TF-CBT. Their replayable format may enhance the learning and reinforcement of therapeutic content while maintaining real-time interaction and professional involvement, although challenges remain in highly interactive components such as trauma narration that rely heavily on in-person communication (Orengo-Aguayo et al., 2018). Digital games may increase children’s engagement through narrative-based, task-oriented, and interactive designs, thereby potentially improving adherence to screening and intervention processes; however, their development requires careful balancing between entertainment value and clinical objectives (Lukka and Palva, 2023). VR can provide children with PTSD a safe and controllable environment to practice emotional regulation and problem-solving skills, offering a highly immersive experience, although its implementation remains constrained by high costs (Park et al., 2019). Overall, these findings suggest that different eHealth modalities may serve complementary roles across distinct stages and needs within pediatric PTSD management.

#### 4.1.3 Social level: real-world feasibility and implementation barriers

From a social perspective, eHealth technologies can overcome geographical barriers, improve access to mental health services, facilitate family involvement, and, to some extent, alleviate pressure on healthcare resources. This review found that most studies reported moderate to high levels of adherence, with generally favorable feasibility and acceptability, consistent with previous findings (Goldzweig et al., 2013; Wong et al., 2020; Schulte et al., 2021). However, these results should be interpreted with caution. Some studies enhanced participation through incentives or free access to interventions, which may have inflated adherence compared with routine real-world service settings. In addition, included studies suggest that when interventions are extended from hospital-based delivery to home-based contexts, completion rates may decrease in the absence of onsite support, indicating that sustained engagement with eHealth interventions is influenced by the surrounding social implementation environment. Factors such as household distractions, limited privacy, unstable internet connectivity, technical difficulties, and user burden may further undermine real-world effectiveness. At a broader societal level, although eHealth may reduce indirect costs such as transportation and caregiver work absence, its initial development, maintenance, and training costs can be substantial. Its scalability and accessibility are therefore influenced by institutional policies and insurance coverage systems (Naslund et al., 2022). Digital inequities may further widen disparities in access to care across regions and families. The included studies show that high-income countries more frequently adopted advanced technologies such as videoconferencing and virtual reality, whereas resource-limited settings primarily relied on mobile-based screening or basic support tools. This pattern suggests that the selection of eHealth approaches should be tailored to local infrastructure, service capacity, and contextual needs. Finally, reporting on adverse events, safety issues, and crisis management procedures remains limited. Given the potential for negative outcomes in digital mental health interventions, more standardized frameworks for safety monitoring, risk detection, and emergency response are warranted. Overall, the long-term development of eHealth in pediatric PTSD management depends not only on technological advancement, but also on coordinated improvements in policy support, implementation infrastructure, and cultural adaptation.

### 4.2 Limitations and future directions

This review included only studies published in English, which may have led to the omission of region-specific evidence reported in other languages and, consequently, to publication bias. Most included evidence originated from high-income countries, while data from low- and middle-income settings were sparse, limiting the global generalizability and applicability of our findings.

Based on the findings of this review, future development of eHealth in pediatric and adolescent PTSD management should focus on three key areas. First, strengthening evidence of clinical effectiveness. The number of randomized controlled trials remains limited, with heterogeneous findings and insufficient long-term follow-up evidence. Future studies should therefore adopt large-sample, rigorously designed investigations to clarify the sustained effects of eHealth interventions, as well as their comparative effectiveness relative to traditional interventions or blended care models. Second, addressing gaps in evidence coverage and enhancing developmental adaptation and individualized design. Current studies are primarily concentrated on the intervention stage, whereas evidence regarding prediction, long-term monitoring, and healthcare professional training remains limited. In addition, most studies focus on school-aged children and adolescents, with insufficient evidence for preschool children. This may be partly attributed to younger children’s greater reliance on caregivers for attention, independent operation, and emotional expression. Future eHealth tools should adopt age-sensitive, individualized, and child-friendly designs, such as incorporating more visual, interactive, and family-coordinated modules for younger children, while enhancing autonomy, privacy protection, and social interaction features for adolescents. Moreover, specific attention should be given to vulnerable populations, including children with autism spectrum disorder, those in foster care, and disaster-exposed populations. In addition, future research should further explore biological and social factors within a biopsychosocial framework to provide a more comprehensive theoretical basis for individualized interventions. Third, emphasizing real-world implementation and system-level support. Although existing studies generally indicate that eHealth is feasible and acceptable, challenges such as variable adherence, technical failures, privacy concerns, and limited digital infrastructure remain prominent. Future research should evaluate the long-term sustainability of eHealth implementation and strengthen safety monitoring mechanisms, reimbursement policies, digital infrastructure development, and digital health competency training for healthcare professionals, so as to promote standardized and sustained implementation in pediatric PTSD care.

## 5 Conclusion

This scoping review indicates that eHealth technologies have been widely applied across all stages of pediatric and adolescent PTSD management, with generally favorable feasibility and acceptability. Different modalities offer distinct functions and application contexts, providing complementary support to traditional mental health services. However, evidence of clinical effectiveness remains limited. Existing research is largely concentrated on school-aged populations and intervention-focused applications, whereas long-term follow-up, predictive monitoring, and vulnerable subgroups remain underrepresented. The field is currently shifting from technology validation toward effectiveness evaluation and system integration. Future research should focus on high-quality studies to support age-specific, individualized, and sustainable implementation of eHealth interventions.

## Footnotes

1
https://osf.io/np4z8/?view_only=71a16577e6de4e8699d905667098d4a3
