# Supplementary material for: eHealth in the management of PTSD in children and adolescents: a scoping review
Source: Front Psychol. 2026 Jul 3;17:1764838. doi: 10.3389/fpsyg.2026.1764838 (PMC13375878; doi:10.3389/fpsyg.2026.1764838)
Supplement: Supplementary file 1 [file Table_1.docx]

Supplementary Material

# Supplementary Table 1. Search Strategy

| Pubmed(175) | |
| --- | --- |
| #1 | ("Child"[Mesh]) OR (Children[Title/Abstract]) OR ("Child, Preschool"[Mesh]) OR (Preschool Child[Title/Abstract]) OR (school child[Title/Abstract]) OR ("Adolescent"[Mesh]) OR (Adolescence[Title/Abstract]) OR (Teen[Title/Abstract]) OR (Teenager[Title/Abstract]) OR (Youth[Title/Abstract]) |
| #2 | ("Telemedicine"[Mesh]) OR ("Virtual Reality"[Mesh]) OR ("Virtual Reality Exposure Therapy"[Mesh]) OR (eHealth[Title/Abstract]) OR (mHealth[Title/Abstract]) OR (Virtual Medicine[Title/Abstract]) OR (Medicine, Virtual[Title/Abstract]) OR (Tele-Referral[Title/Abstract]) OR (Tele Referral[Title/Abstract]) OR (Mobile Health[Title/Abstract]) OR (Health, Mobile[Title/Abstract]) OR (Telehealth[Title/Abstract]) OR (Telecare[Title/Abstract]) OR (Tele-Care[Title/Abstract]) OR (Tele Care[Title/Abstract]) OR (Reality, Virtual[Title/Abstract]) OR (e-health[Title/Abstract]) OR (tele-health[Title/Abstract]) OR (remote monitoring[Title/Abstract]) OR (remote patient monitoring[Title/Abstract]) OR (Wearable monitors[Title/Abstract]) OR (physiological monitoring[Title/Abstract]) OR (Virtual Reality Immersion Therapy[Title/Abstract]) OR (Virtual Reality Therapy[Title/Abstract]) OR (Reality Therapy, Virtual[Title/Abstract]) OR (Therapy, Virtual Reality[Title/Abstract]) |
| #3 | ("Stress Disorders, Post-Traumatic"[Mesh]) OR (Post-Traumatic Stress Disorder[Title/Abstract]) OR (Stress Disorder, Post-Traumatic[Title/Abstract]) OR (PTSD[Title/Abstract]) OR (Post-Traumatic Stress Disorders[Title/Abstract]) OR (Posttraumatic Stress Disorder[Title/Abstract]) OR (Stress Disorder, Posttraumatic[Title/Abstract]) OR (Post Traumatic Stress Disorder[Title/Abstract]) OR (Stress Disorder, Post Traumatic[Title/Abstract]) OR (Post Traumatic Stress Syndrome[Title/Abstract]) OR (PTSS[Title/Abstract]) |
| #4 | #1 AND #2 AND #3 |

| Embase（287） | |
| --- | --- |
| #1 | 'child'/exp or 'child':ab,ti or 'children':ab,ti or 'preschool child'/exp or 'preschool child':ab,ti or 'child, preschool':ab,ti or 'pre-school child':ab,ti or 'pre-school going children':ab,ti or 'pre-schooler':ab,ti or 'Preschooler':ab,ti or 'school child':ab,ti or 'Adolescent':ab,ti or 'Adolescence':ab,ti or 'Teen':ab,ti or 'Teenager':ab,ti or 'Youth':ab,ti |
| #2 | 'telemedicine'/exp OR 'virtual reality'/exp OR 'virtual reality exposure therapy'/exp OR 'telehealth'/exp OR 'Telecare'/exp OR 'remote sensing'/exp OR 'tele medicine':ab,ti OR 'virtual medicine':ab,ti OR 'Telemedicine':ab,ti OR 'virtual reality':ab,ti OR 'e-health':ab,ti OR 'Ehealth':ab,ti OR 'tele-health':ab,ti OR 'Telehealth':ab,ti OR 'virtual reality immersion therapy':ab,ti OR 'VR exposure therapy':ab,ti OR 'VR immersion therapy':ab,ti OR 'VRET':ab,ti OR 'virtual reality exposure therapy':ab,ti OR 'mHealth':ab,ti OR 'Virtual Medicine':ab,ti OR 'Tele-Referral':ab,ti OR 'Mobile Health':ab,ti OR 'e-care':ab,ti OR 'e-health':ab,ti OR 'caree-health':ab,ti OR 'virtual care':ab,ti OR 'virtual health':ab,ti OR 'Carevirtual':ab,ti OR 'Telecare':ab,ti OR 'monitoring, long distance':ab,ti OR 'remote monitoring':ab,ti OR 'remote sensing technology':ab,ti OR 'sensing, remote':ab,ti OR 'telesensing':ab,ti OR 'remote sensing':ab,ti OR 'Wearable monitors':ab,ti OR 'physiological monitoring':ab,ti OR 'web-based':ab,ti |
| #3 | 'posttraumatic stress disorder'/exp OR 'posttraumatic stress disorder':ab,ti OR 'post-traumatic stress':ab,ti OR 'post-traumatic stress disorder':ab,ti OR 'posttraumatic psychic syndrome':ab,ti OR 'posttraumatic psychosis':ab,ti OR 'posttraumatic stress':ab,ti OR 'posttraumatic syndrome':ab,ti OR 'psychosis, posttraumatic':ab,ti OR 'PTSD':ab,ti OR 'stress disorders, post-traumatic':ab,ti OR 'stress disorders, traumatic':ab,ti OR 'stress, posttraumatic':ab,ti OR 'trauma and stressor related disorders':ab,ti OR 'traumatic stresstraumatic stress disorder':ab,ti OR 'traumatic stress disorders':ab,ti OR 'Post Traumatic Stress Syndrome':ab,ti OR 'PTSS':ab,ti |
| #4 | #1 AND #2 AND #3 |

| Cocharne library（97） | |
| --- | --- |
| #1 | MeSH descriptor: [Child] explode all trees |
| #2 | MeSH descriptor: [Child, Preschool] explode all trees |
| #3 | MeSH descriptor: [Adolescent] explode all trees |
| #4 | (Children)：ti,ab,kw OR (Preschool Child)：ti,ab,kw OR (Preschool Children)：ti,ab,kw OR (Children, Preschool)：ti,ab,kw OR (Adolescence)：ti,ab,kw OR (Teen)：ti,ab,kw OR (Teenager)：ti,ab,kw OR (Youth)：ti,ab,kw |
| #5 | MeSH descriptor: [Stress Disorders, Post-Traumatic] explode all trees |
| #6 | (Post-Traumatic Stress Disorder)：ti,ab,kw OR (Stress Disorder, Post-Traumatic)：ti,ab,kw OR (PTSD)：ti,ab,kw OR (Post-Traumatic Stress Disorders)：ti,ab,kw OR (Post Traumatic Stress Disorders)：ti,ab,kw OR (Posttraumatic Stress Disorder)：ti,ab,kw OR (Stress Disorder, Posttraumatic)：ti,ab,kw OR (Post Traumatic Stress Disorder)：ti,ab,kw OR (Stress Disorder, Post Traumatic)：ti,ab,kw OR (Post Traumatic Stress Syndrome)：ti,ab,kw OR (PTSS)：ti,ab,kw |
| #7 | MeSH descriptor: [Telemedicine] explode all trees |
| #8 | MeSH descriptor: [Virtual Reality] explode all trees |
| #9 | MeSH descriptor: [Virtual Reality Exposure Therapy] explode all trees |
| #10 | (eHealth)：ti,ab,kw OR (mHealth)：ti,ab,kw OR (Medicine, Virtual)：ti,ab,kw OR (Virtual Medicine)：ti,ab,kw OR (Tele-Referral)：ti,ab,kw OR (Tele Referral)：ti,ab,kw OR (Mobile Health)：ti,ab,kw OR (Health, Mobile)：ti,ab,kw OR (Telehealth)：ti,ab,kw OR (Telecare)：ti,ab,kw OR (Tele-Care)：ti,ab,kw OR (Tele Care)：ti,ab,kw OR (Reality, Virtual)：ti,ab,kw OR (e-health)：ti,ab,kw OR (tele-health)：ti,ab,kw OR (remote monitoring)：ti,ab,kw OR (remote patient monitoring)：ti,ab,kw OR (Wearable monitors)：ti,ab,kw OR (physiological monitoring)：ti,ab,kw OR (Virtual Reality Immersion Therapy)：ti,ab,kw OR (Virtual Reality Therapy)：ti,ab,kw OR (Reality Therapy, Virtual)：ti,ab,kw OR (Therapy, Virtual Reality)：ti,ab,kw |
| #11 | #1 OR #2 OR #3 OR #4 |
| #12 | #5 OR #6 |
| #13 | #7 OR #8 OR #8 OR #9 OR #10 |
| #14 | #11 AND #12 AND #13 |

| PsyclNFO（37） | |
| --- | --- |
| #1 | SU Child OR SU Child, Preschool OR SU Children OR SU Preschool Child OR SU school child OR SU Adolescent OR SU Adolescence OR SU Teen OR SU Teenager OR SU Youth |
| #2 | SU Telemedicine OR SU Virtual Reality OR SU Virtual Reality Exposure Therapy OR SU eHealth OR SU mHealth OR SU Virtual Medicine OR SU Medicine, Virtual OR SU Tele-Referral OR SU Tele Referral OR SU Mobile Health OR SU Health, Mobile OR SU Telehealth OR SU Telecare OR SU Tele-Care OR SU Tele Care OR SU Reality, Virtual OR SU e-health OR SU tele-health OR SU remote monitoring OR SU remote patient monitoring OR SU Wearable monitors OR SU physiological monitoring OR SU Virtual Reality Immersion Therapy OR SU Virtual Reality Therapy OR SU Reality Therapy, Virtual OR SU Therapy, Virtual Reality |
| #3 | SU Stress Disorders, Post-Traumatic OR SU Post-Traumatic Stress Disorder OR SU PTSD OR SU Post-Traumatic Stress Disorders OR SU Posttraumatic Stress Disorder OR SU Stress Disorder, Posttraumatic OR SU Post Traumatic Stress Disorder OR SU Stress Disorder, Post Traumatic OR SU Post Traumatic Stress Syndrome OR SU PTSS |
| #4 | #1 AND #2 AND #3 |

| **CINAHL**(35) | |
| --- | --- |
|  |  |
| #1 | SU Child OR SU Child, Preschool OR SU Children OR SU Preschool Child OR SU school child OR SU Adolescent OR SU Adolescence OR SU Teen OR SU Teenager OR SU Youth |
| #2 | SU Telemedicine OR SU Virtual Reality OR SU Virtual Reality Exposure Therapy OR SU eHealth OR SU mHealth OR SU Virtual Medicine OR SU Medicine, Virtual OR SU Tele-Referral OR SU Tele Referral OR SU Mobile Health OR SU Health, Mobile OR SU Telehealth OR SU Telecare OR SU Tele-Care OR SU Tele Care OR SU Reality, Virtual OR SU e-health OR SU tele-health OR SU remote monitoring OR SU remote patient monitoring OR SU Wearable monitors OR SU physiological monitoring OR SU Virtual Reality Immersion Therapy OR SU Virtual Reality Therapy OR SU Reality Therapy, Virtual OR SU Therapy, Virtual Reality |
| #3 | SU Stress Disorders, Post-Traumatic OR SU Post-Traumatic Stress Disorder OR SU PTSD OR SU Post-Traumatic Stress Disorders OR SU Posttraumatic Stress Disorder OR SU Stress Disorder, Posttraumatic OR SU Post Traumatic Stress Disorder OR SU Stress Disorder, Post Traumatic OR SU Post Traumatic Stress Syndrome OR SU PTSS |
| #4 | #1 AND #2 AND #3 |

| Scopus(219) |
| --- |
| (((INDEXTERMS(Child)) OR (TITLE-ABS(Children)) OR (INDEXTERMS("Child, Preschool")) OR (TITLE-ABS("Preschool Child")) OR (TITLE-ABS("school child")) OR (INDEXTERMS(Adolescent)) OR (TITLE-ABS(Adolescence)) OR (TITLE-ABS(Teen)) OR (TITLE-ABS(Teenager)) OR (TITLE-ABS(Youth))) AND ((INDEXTERMS(Telemedicine)) OR (INDEXTERMS("Virtual Reality")) OR (INDEXTERMS("Virtual Reality Exposure Therapy")) OR (TITLE-ABS(eHealth)) OR (TITLE-ABS(mHealth)) OR (TITLE-ABS("Virtual Medicine")) OR (TITLE-ABS("Medicine, Virtual")) OR (TITLE-ABS(Tele-Referral)) OR (TITLE-ABS("Tele Referral")) OR (TITLE-ABS("Mobile Health")) OR (TITLE-ABS("Health, Mobile")) OR (TITLE-ABS(Telehealth)) OR (TITLE-ABS(Telecare)) OR (TITLE-ABS(Tele-Care)) OR (TITLE-ABS("Tele Care")) OR (TITLE-ABS("Reality, Virtual")) OR (TITLE-ABS(e-health)) OR (TITLE-ABS(tele-health)) OR (TITLE-ABS("remote monitoring")) OR (TITLE-ABS("remote patient monitoring")) OR (TITLE-ABS("Wearable monitors")) OR (TITLE-ABS("physiological monitoring")) OR (TITLE-ABS("Virtual Reality Immersion Therapy")) OR (TITLE-ABS("Virtual Reality Therapy")) OR (TITLE-ABS("Reality Therapy, Virtual")) OR (TITLE-ABS("Therapy, Virtual Reality")) OR (TITLE-ABS(web-based)))) AND ((INDEXTERMS("Stress Disorders, Post-Traumatic")) OR (TITLE-ABS("Post-Traumatic Stress Disorder")) OR (TITLE-ABS("Stress Disorder, Post-Traumatic")) OR (TITLE-ABS(PTSD)) OR (TITLE-ABS("Post-Traumatic Stress Disorders")) OR (TITLE-ABS("Posttraumatic Stress Disorder")) OR (TITLE-ABS("Stress Disorder, Posttraumatic")) OR (TITLE-ABS("Post Traumatic Stress Disorder")) OR (TITLE-ABS("Stress Disorder, Post Traumatic")) OR (TITLE-ABS("Post Traumatic Stress Syndrome")) OR (TITLE-ABS(PTSS))) |

| Web of science(556) | |
| --- | --- |
| #1 | (((((((((TS=(Child)) OR TS=(Children)) OR TS=(Child, Preschool)) OR TS=(Preschool Child)) OR TS=(school child)) OR TS=(Adolescent)) OR TS=(Adolescence)) OR TS=(Teen)) OR TS=(Teenager)) OR TS=(Youth) |
| #2 | (((((((((((((((((((((((((TS=(Telemedicine)) OR TS=(Virtual Reality)) OR TS=(Virtual Reality Exposure Therapy)) OR TS=(eHealth)) OR TS=(mHealth)) OR TS=(Virtual Medicine)) OR TS=(Medicine, Virtual)) OR TS=(Tele-Referral)) OR TS=(Tele Referral)) OR TS=(Mobile Health)) OR TS=(Health, Mobile)) OR TS=(Telehealth)) OR TS=(Telecare)) OR TS=(Tele-Care)) OR TS=(Tele Care)) OR TS=(Reality, Virtual)) OR TS=(e-health)) OR TS=(tele-health)) OR TS=(remote monitoring)) OR TS=(remote patient monitoring)) OR TS=(Wearable monitors)) OR TS=(physiological monitoring)) OR TS=(Virtual Reality Immersion Therapy)) OR TS=(Virtual Reality Therapy)) OR TS=(Reality Therapy, Virtual)) OR TS=(Therapy, Virtual Reality) |
| #3 | ((((((((((TS=(Stress Disorders, Post-Traumatic)) OR TS=(Post-Traumatic Stress Disorder)) OR TS=(Stress Disorder, Post-Traumatic)) OR TS=(PTSD)) OR TS=(Post-Traumatic Stress Disorders)) OR TS=(Posttraumatic Stress Disorder)) OR TS=(Stress Disorder, Posttraumatic)) OR TS=(Post Traumatic Stress Disorder)) OR TS=(Stress Disorder, Post Traumatic)) OR TS=(Post Traumatic Stress Syndrome)) OR TS=(PTSS) |
| #4 | #1 AND #2 AND #3 |

## **Supplementary Table 2. Mixed method appraisal tool risk or bias rating scores.**

| Studies | 1.1 | 1.2 | 1.3 | 1.4 | 1.5 | 2.1 | 2.2 | 2.3 | 2.4 | 2.5 | 3.1 | 3.2 | 3.3 | 3.4 | 3.5 | 4.1 | 4.2 | 4.3 | 4.4 | 4.5 | 5.1 | 5.2 | 5.3 | 5.4 | 5.5 | Score(%) |
| --- | --- | --- | --- | --- | --- | --- | --- | --- | --- | --- | --- | --- | --- | --- | --- | --- | --- | --- | --- | --- | --- | --- | --- | --- | --- | --- |
| Price et al., 2015 |  |  |  |  |  | U | U | N | U | Y |  |  |  |  |  |  |  |  |  |  |  |  |  |  |  | 20% |
| Marsac et al., 2011 |  |  |  |  |  |  |  |  |  |  | Y | Y | Y | N | Y |  |  |  |  |  |  |  |  |  |  | 80% |
| Piqueras et al., 2021 |  |  |  |  |  |  |  |  |  |  |  |  |  |  |  | Y | Y | Y | U | Y |  |  |  |  |  | 80% |
| Jaycox et al., 2019 |  |  |  |  |  |  |  |  |  |  | Y | Y | N | U | Y |  |  |  |  |  |  |  |  |  |  | 60% |
| Omidvar Eshkalak et al., 2024 |  |  |  |  |  |  |  |  |  |  | Y | Y | Y | N | Y |  |  |  |  |  |  |  |  |  |  | 80% |
| Kassam-Adams et al., 2019 |  |  |  |  |  |  |  |  |  |  |  |  |  |  |  | Y | Y | Y | U | Y |  |  |  |  |  | 80% |
| Martin et al., 2023 | Y | Y | Y | Y | Y |  |  |  |  |  |  |  |  |  |  | Y | Y | Y | N | Y | Y | Y | Y | Y | Y | 80% |
| Hashemi et al., 2017 |  |  |  |  |  |  |  |  |  |  |  |  |  |  |  | Y | Y | Y | Y | Y |  |  |  |  |  | 100% |
| Stewart et al., 2020 |  |  |  |  |  |  |  |  |  |  | Y | Y | Y | N | Y |  |  |  |  |  |  |  |  |  |  | 80% |
| Heinz et al., 2022 | Y | Y | Y | Y | Y |  |  |  |  |  | Y | Y | N | N | Y |  |  |  |  |  | Y | Y | Y | Y | Y | 60% |
| Wijesekera et al., 2023 |  |  |  |  |  | Y | Y | Y | N | N |  |  |  |  |  |  |  |  |  |  |  |  |  |  |  | 60% |
| Asnaani et al., 2021 |  |  |  |  |  |  |  |  |  |  | Y | Y | Y | N | Y |  |  |  |  |  |  |  |  |  |  | 80% |
| Hoover et al., 2019 |  |  |  |  |  |  |  |  |  |  |  |  |  |  |  | U | N | Y | U | Y |  |  |  |  |  | 40% |
| Villalobos et al., 2023 | Y | Y | Y | Y | Y |  |  |  |  |  |  |  |  |  |  | Y | U | Y | U | Y | Y | Y | Y | U | Y | 60% |
| Kassam-Adams et al., 2016 |  |  |  |  |  | U | N | N | Y | Y |  |  |  |  |  |  |  |  |  |  |  |  |  |  |  | 40% |
| Stewart et al., 2017 |  |  |  |  |  |  |  |  |  |  | Y | Y | Y | N | Y |  |  |  |  |  |  |  |  |  |  | 80% |
| Goslin et al., 2024 |  |  |  |  |  |  |  |  |  |  | Y | Y | Y | N | Y |  |  |  |  |  |  |  |  |  |  | 80% |
| Marsac et al., 2013 |  |  |  |  |  | U | Y | Y | U | Y |  |  |  |  |  |  |  |  |  |  |  |  |  |  |  | 60% |
| Hashemi et al., 2017 |  |  |  |  |  |  |  |  |  |  | Y | Y | Y | N | Y |  |  |  |  |  |  |  |  |  |  | 80% |
| Shenk et al., 2022 |  |  |  |  |  | Y | Y | Y | N | Y |  |  |  |  |  |  |  |  |  |  |  |  |  |  |  | 80% |
| Cox et al., 2010 |  |  |  |  |  | Y | Y | Y | Y | Y |  |  |  |  |  |  |  |  |  |  |  |  |  |  |  | 100% |
| Fein et al., 2010 |  |  |  |  |  |  |  |  |  |  |  |  |  |  |  | Y | Y | Y | U | Y |  |  |  |  |  | 80% |
| Smith et al., 2025 |  |  |  |  |  | Y | U | Y | Y | Y |  |  |  |  |  |  |  |  |  |  |  |  |  |  |  | 80% |
| Prieto et al., 2025 |  |  |  |  |  |  |  |  |  |  | Y | Y | N | N | Y |  |  |  |  |  |  |  |  |  |  | 60% |
| Birkeland et al., 2025 | Y | Y | Y | Y | Y |  |  |  |  |  | Y | Y | N | N | Y |  |  |  |  |  | Y | Y | Y | Y | Y | 60% |
| McDonnell et al., 2025 |  |  |  |  |  |  |  |  |  |  | Y | Y | N | N | Y |  |  |  |  |  |  |  |  |  |  | 60% |

Y: Yes

N: NO

U: Unknow
